# Garlic consumption and colorectal cancer risk in US adults: a large prospective cohort study

**DOI:** 10.3389/fnut.2023.1300330

**Published:** 2023-12-06

**Authors:** Zongze Jiang, Huilin Chen, Ming Li, Wei Wang, Feiwu Long, Chuanwen Fan

**Affiliations:** ^1^Department of Gastrointestinal, Bariatric and Metabolic Surgery, Research Center for Nutrition, Metabolism and Food Safety, West China-PUMC C.C. Chen Institute of Health, West China School of Public Health and West China Fourth Hospital, Sichuan University, Chengdu, China; ^2^Department of Immunology, Institute of Basic Medical Sciences, Chinese Academy of Medical Sciences, School of Basic Medicine, Peking Union Medical College, Beijing, China; ^3^Department of Nutrition, Food Hygiene, and Toxicology, West China School of Public Health and West China Fourth Hospital, Sichuan University, Chengdu, China; ^4^Department of Oncology and Department of Biomedical and Clinical Sciences, Linköping University, Linköping, Sweden

**Keywords:** garlic, colorectal cancer, adults, cohort study, epidemiology

## Abstract

**Objective:**

To clarify the inconsistent findings of epidemiological studies on the association between dietary garlic consumption and colorectal cancer (CRC) incidence, by prospectively assessing the association in a large US population.

**Methods:**

Data of 58,508 participants (aged 55–74) from the Prostate, Lung, Colorectal, and Ovarian (PLCO) Cancer Screening Trial were analyzed. Dietary data were collected using a validated questionnaire. Multivariable Cox regression analysis determined hazard ratio (HR) and 95% confidence interval (CI). Restricted cubic spline regression was used to investigate the non-linear relationship, and subgroup analysis was conducted to examine potential effect modifiers.

**Results:**

During a median follow-up of 12.05 years, 782 CRC cases were documented, including 456 proximal colon cancer cases, 322 distal CRC cases, and 4 CRC cases with an unknown site. Moderate dietary garlic consumption was significantly associated with a reduced risk of overall CRC (HR_*quintile* 3*vs*. 1_: 0.70, 95% CI: 0.54 to 0.91, *p* = 0.007, *P* for trend: 0.434), exhibiting a U-shaped dose-response pattern, and also with overall CRC in males in the stratified Cox regression model (Model 2: HR_*quintile* 3*vs*. 1_: 0.57, 95% CI: 0.40 to 0.81, *p* = 0.002), but not in females. The protective association was more pronounced in men, Caucasian, and those with lower alcohol consumption. Notably, these protective effects were observed for overall distal CRC (HR_*quintile* 3*vs*. 1_: 0.62, 95% CI: 0.42 to 0.93, *p* = 0.021; and HR_*quintile* 4*vs*. 1_: 0.63, 95% CI: 0.43 to 0.92, *p* = 0.018, *P* for trend: 0.208); and for distal CRC in males (HR_*quintile* 3*vs*. 1_: 0.40, 95% CI: 0.22 to 0.71, *p* = 0.002, *P* for trend: 0.696), but not for proximal CRC.

**Conclusion:**

Moderate consumption of dietary garlic is associated with a decreased CRC risk in the US population, with variations based on CRC anatomic subsites. Further in-depth prospective studies are needed to validate these findings in different populations and to explore subsites-specific associations.

## Introduction

Colorectal cancer (CRC) is the second most common cause of cancer death in the United States, with an estimated 153,020 new cases and 52,550 fatalities expected in 2023, including a concerning number among those under 50 years old (1). In addition to genetic factors, over half of CRC cases are linked to modifiable lifestyle risk factors, including obesity, physical inactivity, alcohol drinking, and smoking (2). Also, diet high in plant-based foods has been associated with a reduced likelihood of developing the disease (3). Specifically, garlic (Allium sativum L.) has shown an inverse association with CRC risk in case-control studies, although findings from cohort studies remain controversial (4, 5).

Garlic, a widely consumed non-digestible vegetable rich in organosulfur compounds and flavonoids, might be related with a lower risk of CRC through multiple mechanisms, including inhibition of carcinogen-induced DNA adduct formation, cell proliferation, angiogenesis, and inhibition of Cox-2 expression (6–8). It is notable that although previous studies have demonstrated a generally inverse association of garlic consumption with all-cause mortality (9) and various cancer sites (10–15), including colorectal cancer, these findings are inconsistent. While the recent meta-analysis (4, 5, 16–19) and case-controlled studies (20–22) support this inverse association, some meta-analysis (23–25) and prospective cohorts (26, 27) showed no such association.

Given that the existing evidence is largely derived from case-control studies, which are susceptible to recall bias and unable to establish a time-based association. Moreover, there haven’t been any prospective cohorts to assess the non-linear relationship of garlic consumption with CRC over a period of time in the US population. To fill this research gap, we conducted a prospective cohort study using the data from the Prostate, Lung, Colorectal, and Ovarian (PLCO) cancer screening trial to comprehensively explore the association between dietary garlic consumption and CRC incidence.

## Materials and methods

### Data source and study population

The PLCO cancer screening trial was a large-scale, multicenter randomized controlled study sponsored by the United States National Cancer Institute (NCI) to determine whether specific screening examinations reduce mortality from PLCO cancers in US adults aged 55–74 years. Details of the study design and methodology have been described elsewhere (28). Briefly, a total of 154,887 participants including 76,678 men and 78,209 women were recruited between 1993 and 2001 from ten screening centers (Washington, Denver, Marshfield, Detroit, Minneapolis, Birmingham, Pittsburgh, Honolulu, Salt Lake City, and St Louis) across the United States. Upon enrollment, they were randomly assigned to either a control group or an intervention group. The PLCO trial was conducted with approval from the Institutional Review Boards of the National Cancer Institute (NCI). Each of the ten study centers obtained approval from their local Institutional Review Boards. All participants provided written informed consent before enrollment in the study.

**FIGURE 1:**
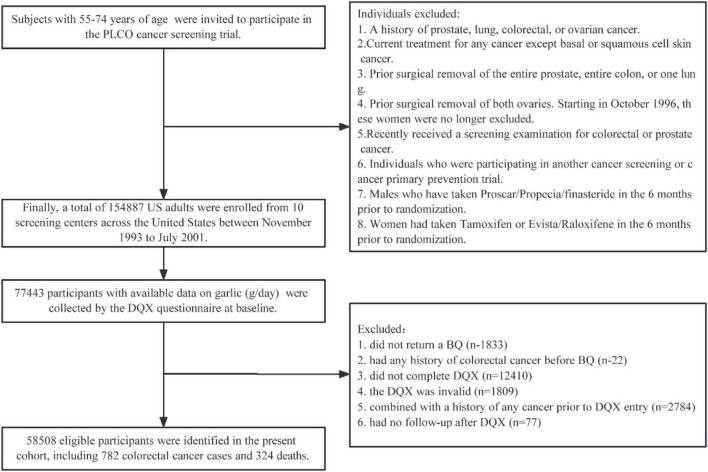
The flowchart of subjects identified in our study. BQ, baseline questionnaire; DQX, dietary questionnaire, PLCO, Prostate, Lung, Colorectal, and Ovarian.

In the present study, 77,443 participants with available data on garlic (g/day) consumption in the intervention arm were collected from the DQX questionnaire at baseline (T0). Sequentially, participants were further excluded if they (1) did not return the baseline questionnaire (*n* = 1,833) or had any history of CRC before the baseline questionnaire (*n* = 22); (2) had an incomplete DQX questionnaire (*n* = 12,410) or an invalid DQX that was missing the completion date, was completed before the date of death, had ≥ 8 missing frequency responses, or indicated extremely high or low-calorie intake (i.e., top 1% or bottom 1%) (*n* = 1,809); (3) had a history of any cancer before DQX entry (*n* = 2,874); or (4) had no follow-up time after the DQX (*n* = 77). Ultimately, our cohort consisted of 58,508 eligible participants (Figure 1).

### Data collection

Participants in the study completed a comprehensive baseline questionnaire, providing self-reported data on demographics, lifestyle factors, and medical history, including sex, race, trial arm, body mass index (BMI), educational level, marital status, aspirin use, cigarette smoking, family history of CRC, history of colon comorbidities, history of colorectal polyps, and diabetes history. BMI was calculated as weight in kilograms divided by the square of height in meters. Dietary data at baseline (T0), encompassing alcohol consumption, dietary energy intake, and dietary foods or nutrient intake, were collected using a 137-item self-administered food-frequency questionnaire known as the DQX. The DQX questionnaire was derived from 2 previously validated food frequency questionnaire (FFQs) developed for epidemiologic and clinical use. It included items from the 61-item semiquantitative Willett FFQ that have been shown to provide adequate information on individual nutrient intake over 1 year and other items from the Block FFQ developed from the Second National Health and Nutrition Examination Survey (29, 30). During the dietary survey, participants were instructed to recall the average frequency of consuming each food item listed in an FFQ over the past year. Dietary intake of energy and nutrients was calculated by multiplying the amount of energy and nutrients in the standard portion size of each food item by the reported frequency. The values were then summed across all food items, utilizing the United States Department of Agriculture’s 1994–1996 Continuing Survey of Food Intakes by Individuals or the widely employed Nutrition Data Systems for Research nutrient database (31). Healthy Eating Index-2005, a metric for assessing diet quality, was calculated following the methodology outlined in the literature (32). Physical activity levels were assessed using the DQX questionnaire, specifically quantifying hours engaged in vigorous activities at present.

### Ascertainment of colorectal cancer

The main outcome measure of the study was the occurrence of CRC, determined through annual reviews of participants’ medical records, which provided updates on cancer diagnoses, including the date of detection and cancer site. A standardized form was used to review relevant medical records to ensure the accuracy of the reported cancer cases. Cases and their anatomical locations were confirmed by study physicians who were blinded to participants’ risk factors. CRC was defined according to the International Classification of Diseases for Oncology (ICD-O-2 codes: colon cancer: C18, and rectal cancer: C19-C20). Proximal colorectal cancers encompassed cecum, appendix, ascending colon, hepatic flexure, transverse colon, and splenic flexure colon cancer, while distal CRC included descending cancer, sigmoid colon cancer, rectosigmoid junction cancer, and rectal cancer. The follow-up duration was calculated from the date of DQX completion until the first instance of CRC diagnosis, participant dropout, CRC-related death, or the end of the follow-up period, which extended until 31 December 2009.

### Statistical analysis

To address missing data for twelve covariates (Supplementary Table 1) and enhance statistical power while minimizing potential biases, we utilized multiple imputations with the random forest algorithm (R package “missRanger”) to impute twelve covariates with missing data, assuming that the missing data were random. The imputed data set included all variables used in the statistical analyses. Furthermore, we conducted additional analyses of participants with complete data for comparison purposes.

We employed Cox proportional hazards regression to estimate hazard ratios (HRs) and 95% confidence intervals (CIs) to assess the association between dietary garlic consumption and CRC incidence, using follow-up time as the underlying time metric. Garlic intake was adjusted for energy using the residual method (33) and categorized into quintiles, with the lowest quintile serving as the reference group. To assess linear trends in risk estimates across quintiles of energy-adjusted garlic consumption, we assigned the median value of each quintile to the corresponding participants, creating an ordinal variable. This ordinal variable was treated as a continuous variable in regression models, and its significance in indicating linear trends was assessed using the Wald test to obtain the associated *p*-value. In Cox models, we selected covariates entering into multivariable analyses based on our casual knowledge and used the directed acyclic graph to visualize the relationship among exposure, outcome, and potential confounders (DAGitty version 3.0; www.dagitty.net/) (Supplementary Figure 1). A total of 9 potential confounders were identified, and we verified the proportional hazard assumption by the Schoenfeld residual test (all *p*-values for the global test > 0.05; listed in Supplementary Table 2). Among them, the variable “sex” violated the PH assumption, therefore, we also conducted the stratified Cox regression to control the time-varying effect of “sex.” Furthermore, we applied the Marginal Structural Model (MSM) to adjust the potential time-varying dietary exposure or variables in the Cox regression models (34–36). Specifically, the Crude model adjusted for none; Model 1 adjusted for age (years), and sex (male vs. female). Model 2 adjusted for age (years), sex (male vs. female), race (white, non-Hispanic vs. black, non-Hispanic vs. Hispanic vs. others), physical activity (none vs. ≤ 1 h/week vs. ≥ 2 h/week), diabetes (no vs. yes), cigarette smoking (never vs. current vs. former), BMI (kg/m^2^), alcohol consumption (g/day), and energy from diet (kcal/day); Model 3 adjusted the covariates in Model 2 using the Marginal Structural Model; Model 4 adjusted for all variables at baseline, including age (years), sex (male vs. female), marital status (married vs. unmarried), race (white, non-Hispanic vs. black, non-Hispanic vs. Hispanic vs. others), education level (≤ some college vs. college graduate vs. postgraduate), physical activity (none vs. ≤ 1 h/week vs. ≥ 2 h/week), multivitamin use (no vs. yes), aspirin use (no vs. yes), diabetes (no vs. yes), cigarette smoking (never vs. current vs. former), pack-years (continuous), BMI (kg/m^2^), family history of colorectal cancer (no vs. yes vs. possibly), alcohol consumption (g/day), history of colorectal polyps (no vs. yes), history of colon comorbidities (no vs. yes), and energy from diet (kcal/day); Model 5 adjusted all the covariates in Model 4 using the Marginal Structural Model.

We conducted subgroup analyses to evaluate whether the association between garlic consumption and CRC incidence was influenced by age (< median vs. ≥ median), sex (male vs. female), race (white, non-Hispanic vs. black, non-Hispanic vs. others), BMI (< 25 kg/m^2^ vs. ≥ 25 kg/m^2^), smoking status (current/former vs. never), and alcohol consumption (no/light/moderate vs. heavy). For alcohol consumption, we categorized it as light, moderate, and heavy. Light alcohol consumption was defined as up to 6 g/day. Moderate consumption was defined as more than 6–28 g/day for males and more than 6–14 g/day for females, and heavy consumption was defined as more than 28 g/day for males and more than 14 g/day for females, respectively (37). To assess the modification effect, we used a likelihood ratio test by comparing models with and without interaction terms to obtain a *P* interaction. Furthermore, we also categorized the garlic consumption into tertiles and repeated the above-mentioned subgroup analyses to minimize the bias from small case numbers of each stratum.

A series of wide-range sensitivity analyses were conducted as following steps: (1) excluded participants with extreme energy intake from diet (< 800 or > 4,000 kcal/day for men and < 500 or > 3,500 kcal/day for women); (2) excluded participants with a history of diabetes; (3) excluded participants with extreme BMI (top 1% and bottom 1% of BMI); (4) excluded participants within the first 2 years of follow-up; (5) repeated analysis for participants with complete data; (6) additionally adjusting for the Healthy Eating Index-2015 to examine whether the observed correlation was influenced by diet quality. We employed restricted cubic spline functions with 4 knots (5, 35, 65, and 95th percentiles) to explore potential non-linear relationships between energy-adjusted dietary garlic consumption and CRC incidence. It is important to note that participants with dietary intakes below the 1st percentile or above the 99th percentile were excluded to minimize potential bias from extreme values in the dose-response analyses. Furthermore, we assessed the significance of non-linearity by testing the null hypothesis that the regression coefficient of the second spline was equal to zero. All statistical analyses were performed using R software (version 4.2.1), with a two-tailed significance level set at *P* < 0.05.

## Results

### Participants’ baseline characteristics

We identified a total of 58,508 participants for this study. During a median follow-up of 12.05 years, there were 782 cases of colorectal cancer and 324 deaths, Energy-adjusted dietary garlic consumption ranged from −1.15 to 11.68 g/day (median value: 0.42 g/day), whereas the unadjusted dietary garlic consumption ranged from 0 to 11.33 g/day (median value: 0.34 g/day). Table 1 summarizes the baseline characteristics of these participants by quintiles of energy-adjusted garlic consumption.

**TABLE 1:** Baseline characteristics of the study population according to quintiles of energy-adjusted garlic consumption (g/day) in 58,508 participants.

	Quintiles of energy-adjusted garlic consumption (g/day)						
Variables	Overall	Quintile 1 (≤ 0.082)	Quintile 2 (0.082–0.301)	Quintile 3 (0.301–0.603)	Quintile 4 (0.603–1.481)	Quintile 5 (> 1.481)	*p*
Number of participants	58,508	11,704	11,678	11,736	11,687	11,703	
Age at DQX entry (years)	62.00 [58.00, 67.00]	62.00 [58.00, 67.00]	63.00 [59.00, 68.00]	62.00 [58.00, 67.00]	62.00 [58.00, 66.00]	62.00 [58.00, 66.00]	< 0.001
**Sex (%)**							
Male	30,110 (51.46)	8,365 (71.47)	5,614 (48.07)	4,583 (39.05)	4,380 (37.48)	7,168 (61.25)	< 0.001
Female	28,398 (48.54)	3,339 (28.53)	6,064 (51.93)	7,153 (60.95)	7,307 (62.52)	4,535 (38.75)	
**Physical activity (%)**							
None	8,924 (15.25)	2,066 (17.65)	1,823 (15.61)	1,873 (15.96)	1,557 (13.32)	1,605 (13.71)	< 0.001
≤ 1 h/week	17,317 (29.60)	3,437 (29.37)	3,662 (31.36)	3,646 (31.07)	3,402 (29.11)	3,170 (27.09)	
≥ 2 h/week	32,267 (55.15)	6,201 (52.98)	6,193 (53.03)	6,217 (52.97)	6,728 (57.57)	6,928 (59.20)	
Alcohol consumption (g/day)	1.42 [0.21, 11.13]	1.50 [0.20, 15.31]	0.85 [0.04, 7.58]	0.87 [0.11, 7.39]	1.74 [0.28, 12.88]	2.77 [0.30, 17.77]	< 0.001
**Multi-vitamin use (%)**							
No	28,904 (49.40)	6,466 (55.25)	6,092 (52.17)	5,720 (48.74)	5,326 (45.57)	5,300 (45.29)	< 0.001
Yes	29,604 (50.60)	5,238 (44.75)	5,586 (47.83)	6,016 (51.26)	6,361 (54.43)	6,403 (54.71)	
**Race (%)**							
White, non-Hispanic	53,149 (90.84)	11,041 (94.34)	10,956 (93.82)	10,762 (91.70)	10,501 (89.85)	9,889 (84.50)	< 0.001
Black, non-Hispanic	2,245 (3.84)	414 (3.54)	406 (3.48)	456 (3.89)	378 (3.23)	591 (5.05)	
Hispanic	887 (1.52)	99 (0.85)	108 (0.92)	136 (1.16)	205 (1.75)	339 (2.90)	
Others	2,227 (3.81)	150 (1.28)	208 (1.78)	382 (3.25)	603 (5.16)	884 (7.55)	
**Education (%)**							
≤ Some college	37,468 (64.04)	7,627 (65.17)	7,822 (66.98)	7,729 (65.86)	7,247 (62.01)	7,043 (60.18)	< 0.001
College graduate	10,206 (17.44)	1,999 (17.08)	1,965 (16.83)	1,991 (16.96)	2,125 (18.18)	2,126 (18.17)	
Postgraduate	10,834 (18.52)	2,078 (17.75)	1,891 (16.19)	2,016 (17.18)	2,315 (19.81)	2,534 (21.65)	
**Marital status (%)**							
Married	45,847 (78.36)	9,497 (81.14)	9,119 (78.09)	8,908 (75.90)	9,084 (77.73)	9,239 (78.95)	< 0.001
Unmarried	12,661 (21.64)	2,207 (18.86)	2,559 (21.91)	2,828 (24.10)	2,603 (22.27)	2,464 (21.05)	
**Cigarette smoking (%)**							
Never	27,409 (46.85)	5,558 (47.49)	6,016 (51.52)	5,831 (49.68)	5,347 (45.75)	4,657 (39.79)	< 0.001
Current	5,745 (9.82)	1,248 (10.66)	1,038 (8.89)	1,087 (9.26)	1,141 (9.76)	1,231 (10.52)	
Former	25,354 (43.33)	4,898 (41.85)	4,624 (39.60)	4,818 (41.05)	5,199 (44.49)	5,815 (49.69)	
Pack years	3.50 [0.00, 31.00]	3.50 [0.00, 34.00]	0.00 [0.00, 26.00]	0.50 [0.00, 27.00]	4.50 [0.00, 30.00]	10.00 [0.00, 36.00]	< 0.001
**Family history of colorectal cancer (%)**							
No	50,859 (86.93)	10,142 (86.65)	10,095 (86.44)	10,164 (86.61)	10,234 (87.57)	10,224 (87.36)	< 0.001
Yes	6,074 (10.38)	1,175 (10.04)	1,245 (10.66)	1,292 (11.01)	1,205 (10.31)	1,157 (9.89)	
Possibly	1,575 (2.69)	387 (3.31)	338 (2.89)	280 (2.39)	248 (2.12)	322 (2.75)	
BMI (kg/m^2^)	26.58 [24.10, 29.69]	27.12 [24.63, 29.99]	26.54 [24.03, 29.53]	26.36 [23.73, 29.29]	26.50 [23.72, 29.62]	26.61 [24.21, 29.95]	< 0.001
**Aspirin use**							
No	30,875 (52.77)	6,108 (52.19)	6,235 (53.39)	6,438 (54.86)	6,233 (53.33)	5,861 (50.08)	< 0.001
Yes	27,633 (47.23)	5,596 (47.81)	5,443 (46.61)	5,298 (45.14)	5,454 (46.67)	5,842 (49.92)	
**History of colon comorbidities (%)**							
No	57,701 (98.62)	11,544 (98.63)	11,517 (98.62)	11,560 (98.50)	11,522 (98.59)	11,558 (98.76)	0.55
Yes	807 (1.38)	160 (1.37)	161 (1.38)	176 (1.50)	165 (1.41)	145 (1.24)	
**Diabetes (%)**							
No	54,326 (92.85)	10,861 (92.80)	10,847 (92.88)	10,900 (92.88)	10,978 (93.93)	10,740 (91.77)	< 0.001
Yes	4,182 (7.15)	843 (7.20)	831 (7.12)	836 (7.12)	709 (6.07)	963 (8.23)	
**History of colorectal polyps (%)**							
No	54,389 (92.96)	10,805 (92.32)	10,845 (92.87)	10,991 (93.65)	10,913 (93.38)	10,835 (92.58)	< 0.001
Yes	4,119 (7.04)	899 (7.68)	833 (7.13)	745 (6.35)	774 (6.62)	868 (7.42)	
Healthy Eating Index-2015	67.29 [60.87, 73.07]	63.94 [57.43, 70.01]	66.91 [60.56, 72.61]	67.61 [61.53, 73.16]	68.80 [62.60, 74.24]	69.00 [62.83, 74.43]	< 0.001
Total energy from diet (kcal/day)	1,910.11 [1,474.11, 2,477.65]	2,599.20 [2,232.25, 3,132.81]	1,731.86 [1,506.60, 1,976.01]	1,413.49 [1,108.17, 1,900.40]	1,745.98 [1,375.35, 2,233.49]	2,071.48 [1,613.15, 2,584.43]	< 0.001

DQX: dietary questionnaire; “Others” refers to Hispanic, Asian, Pacific Islander, or American Indian; BMI, body mass index; Values are presented as median [interquartile range] for continuous variables or counts (percentage) for categorical variables, respectively.

Compared to participants with the lowest level of garlic intake, those with the highest quintile for garlic intake were generally more physically active, consumed more alcohol, had higher educational levels, reported more pack-years of smoking, and also had a lower overall caloric intake from diet. Meanwhile, participants with moderate garlic intake (quintile 2, quintile 3, and quintile 4) were predominantly females, consumed less alcohol, had a lower proportion of current smokers, and also lower dietary energy intake.

### Garlic consumption and CRC incidence

In this study, which tracked 676,471 person-years of follow-up, we identified 782 CRC cases, including 456 proximal colon cancer, 322 distal CRC (comprising distal colon and rectal cancer), and 4 CRC cases with an unspecific location. The overall incidence rate was 1.16 cases per 1,000 person-years.

To investigate the relationship between dietary garlic consumption and the incidence of overall colorectal cancer, as well as its subsites, we employed multivariable Cox regression models as shown in Table 2. As for overall colorectal cancer, the full-adjusted model (Model 2) indicated that those in the moderate consumption category (quintile 3) were associated with a 30% lower risk of overall CRC incidence (HR_*quintile* 3*vs*. 1_: 0.70, 95% CI: 0.54 to 0.91, *p* = 0.007, *P* for trend: 0.434) compared to the lowest consumption (quintile 1) of energy-adjusted dietary garlic. Considering the potential time-varying effect from the dietary exposure and variables, we applied the Marginal Structural Mode to further analyses and found a similar inverse association in Model 3 (HR_*quintile* 3*vs*. 1_: 0.74, 95% CI: 0.59 to 0.94, *p* = 0.013). In addition, we included all variables at baseline in Model 4 (PH assumption showed global test < 0.05) as a comparative analysis versus Model 2 and used the MSM model to control time-varying variables, but we didn’t observe a significant change (Model 4: HR_*quintile* 3*vs*. 1_: 0.71, 95% CI: 0.55 to 0.92, *p* = 0.009; Model 5: HR_*quintile* 3*vs*. 1_: 0.73, 95% CI: 0.58 to 0.93, *p* = 0.009). Next, we conducted the stratified Cox regression by sex, and found a consistent inverse association in males (Model 2: HR_*quintile* 3*vs*. 1_: 0.57, 95% CI: 0.40 to 0.81, *p* = 0.002), but not in females. Similarly, we also observed the inverse association between moderate consumption of garlic and overall distal CRC (HR_*quintile* 3*vs*. 1_: 0.62, 95% CI: 0.42 to 0.93, *p* = 0.021; and HR_*quintile* 4*vs*. 1_: 0.63, 95% CI: 0.43 to 0.92, *p* = 0.018, *P* for trend: 0.208); and distal CRC in males (HR_*quintile* 3*vs*. 1_: 0.40, 95% CI: 0.22 to 0.71, *p* = 0.002, *P* for trend: 0.696), but not in females. In contrast, we detected a suggestive but not significant inverse association between garlic consumption and proximal colon cancer, both in males and females.

**TABLE 2:** Association between energy-adjusted dietary garlic consumption (g/day) and colorectal cancer incidence in the PLCO cancer screening trial.

	Quintiles of energy-adjusted garlic consumption, range (median), g/day					
Variables	≤ 0.082 (−0.087)	0.082–0.301 (0.197)	0.301–0.603 (0.415)	0.603–1.481 (0.934)	> 1.481 (2.424)	*P* interaction^a^
**Colorectal cancer^b^**						0.01
No. of participants	11,704	11,678	11,736	11,687	11,703	
Cases	179	175	131	143	154	
Person-years	136,102.2	135,645.7	135,497.5	135,424.8	133,800.7	
Incidence rate^c^	1.315188	1.290125	0.9668076	1.055937	1.150965	
Crude model^d^	1 (Ref)	0.98 (0.80–1.21)	0.74 (0.59–0.92)	0.80 (0.64–1.00)	0.87 (0.70–1.08)	
Model 1	1 (Ref)	1.01 (0.81–1.24)	0.80 (0.63–1.00)	0.91 (0.72–1.14)	0.93 (0.75–1.16)	
Model 2	1 (Ref)	0.92 (0.73–1.16)	0.71 (0.55–0.92)	0.84 (0.66–1.07)	0.87 (0.69–1.09)	
Model 3 (DAG)	1 (Ref)	0.92 (0.73–1.15)	0.70 (0.54–0.91)	0.82 (0.64–1.04)	0.85 (0.67–1.06)	
Model 4 (MSM-DAG)	1 (Ref)	0.98 (0.79–1.22)	0.74 (0.59–0.94)	0.82 (0.65–1.03)	0.85 (0.67–1.08)	
Model 5 (MSM-full)	1 (Ref)	0.97 (0.78–1.21)	0.73 (0.58–0.93)	0.82 (0.65–1.04)	0.86 (0.67–1.09)	
**Males**						
No. of participants	6,018	6,031	6,017	6,021	6,023	
cases (person-years)	137 (97,510.2)	95 (65,339.89)	47 (53,190.42)	66 (50,875.31)	109 (81,663.13)	
Crude model	Ref	1.03 (0.80–1.34)	0.63 (0.45–0.88)	0.92 (0.69–1.24)	0.95 (0.74–1.22)	
Model 3 (DAG)	Ref	0.94 (0.70–1.25)	0.57 (0.40–0.81)	0.89 (0.65–1.21)	0.91 (0.70–1.19)	
Model 4 (MSM-DAG)	Ref	1.06 (0.81–1.39)	0.64 (0.45–0.89)	0.91 (0.67–1.24)	0.91 (0.70–1.19)	
**Females**						
No. of participants	5,671	5,692	5,673	5,681	5,681	
cases (person-years)	42 (38,592.03)	80 (70,305.85)	84 (82,307.07)	77 (84,549.44)	45 (52,137.62)	
Crude model	Ref	1.05 (0.72–1.52)	0.94 (0.65–1.36)	0.84 (0.57–1.22)	0.79 (0.52–1.21)	
Model 3 (DAG)	Ref	0.87 (0.58–1.32)	0.76 (0.49–1.18)	0.73 (0.48–1.11)	0.70 (0.45–1.10)	
Model 4 (MSM-DAG)	Ref	0.99 (0.67–1.46)	0.92 (0.63–1.36)	0.84 (0.56–1.25)	0.74 (0.46–1.19)	
Proximal colon cancer						0.22
No. of participants	11,704	11,678	11,736	11,687	11,703	
Cases	98	102	76	90	90	
Person-years	136,102.2	135,645.7	135,497.5	135,424.8	133,800.7	
Incidence rate^c^	0.720047	0.7519587	0.560896	0.6645757	0.672642	
Crude model	1 (Ref)	1.05 (0.79–1.38)	0.78 (0.58–1.05)	0.93 (0.70–1.23)	0.94 (0.70–1.25)	
Model 1	1 (Ref)	1.04 (0.79–1.38)	0.82 (0.61–1.12)	1.03 (0.77–1.38)	1.00 (0.75–1.34)	
Model 2	1 (Ref)	0.98 (0.73–1.34)	0.77 (0.54–1.08)	0.98 (0.72–1.35)	0.97 (0.72–1.31)	
Model 3 (DAG)	1 (Ref)	0.98 (0.72–1.33)	0.76 (0.54–1.07)	0.98 (0.71–1.34)	0.96 (0.71–1.30)	
Model 4 (MSM-DAG)	1 (Ref)	1.03 (0.77–1.37)	0.81 (0.59–1.10)	0.97 (0.72–1.31)	0.96 (0.70–1.31)	
Model 5 (MSM-full)	1 (Ref)					
**Males**						
No. of participants	6,018	6,031	6,017	6,021	6,023	
Cases (person-years)	73 (97,510.2)	51 (65,339.89)	30 (53,190.42)	41 (50,875.31)	59 (81,663.13)	
Crude model	1 (Ref)	1.04 (0.73–1.49)	0.75 (0.49–1.15)	1.08 (0.74–1.58)	0.97 (0.69–1.37)	
Model 3 (DAG)	1 (Ref)	0.98 (0.66–1.44)	0.72 (0.46–1.14)	1.12 (0.75–1.68)	1.01 (0.70–1.44)	
Model 4 (MSM-DAG)	1 (Ref)	1.05 (0.73–1.52)	0.79 (0.51–1.22)	1.11 (0.74–1.65)	1.00 (0.70–1.44)	
**Females**						
No. of participants	5,671	5,692	5,673	5,681	5,681	
Cases (person-years)	25 (38,592.03)	51 (70,305.85)	46 (82,307.07)	49 (84,549.44)	31 (52,137.62)	
Crude model	1 (Ref)	1.12 (0.69–1.81)	0.86 (0.53–1.40)	0.90 (0.55–1.45)	0.92 (0.54–1.56)	
Model 3 (DAG)	1 (Ref)	0.95 (0.57–1.61)	0.73 (0.41–1.30)	0.82 (0.48–1.39)	0.85 (0.48–1.49)	
Model 4 (MSM-DAG)	1 (Ref)	1.01 (0.60–1.70)	0.84 (0.50–1.43)	0.90 (0.53–1.52)	0.86 (0.47–1.58)	
**Distal colorectal cancer**						0.002
No. of participants	11,704	11,678	11,736	11,687	11,703	
Cases	79	72	55	52	64	
Person-years	136,102.2	135,645.7	135,497.5	135,424.8	133,800.7	
Incidence rate^c^	0.580446	0.5307944	0.4059116	0.3839771	0.4783232	
Crude model	1 (Ref)	0.91 (0.66–1.26)	0.70 (0.49–0.98)	0.66 (0.46–0.94)	0.82 (0.59–1.14)	
Model 1	1 (Ref)	0.98 (0.71–1.35)	0.78 (0.55–1.11)	0.76 (0.53–1.09)	0.87 (0.62–1.21)	
Model 2	1 (Ref)	0.84 (0.59–1.20)	0.64 (0.43–0.95)	0.66 (0.45–0.97)	0.76 (0.53–1.08)	
Model 3 (DAG)	1 (Ref)	0.83 (0.58–1.18)	0.62 (0.42–0.93)	0.63 (0.43–0.92)	0.73 (0.51–1.03)	
Model 4 (MSM-DAG)	1 (Ref)	0.94 (0.67–1.32)	0.68 (0.47–0.97)	0.62 (0.42–0.89)	0.73 (0.51–1.06)	
Model 5 (MSM-full)	1 (Ref)	0.94 (0.67–1.32)	0.68 (0.47–0.96)	0.64 (0.44–0.91)	0.77 (0.53–1.12)	
**Males**						
No. of participants	6,018	6,031	6,017	6,021	6,023	
Cases (person-years)	50 (70,079.92)	37 (70,278.04)	35 (69,928.28)	36 (69,785.5)	40 (68,507.21)	
Crude model	1 (Ref)	1.04 (0.71–1.53)	0.49 (0.29–0.84)	0.73 (0.46–1.17)	0.94 (0.65–1.36)	
Model 3 (DAG)	1 (Ref)	0.88 (0.58–1.35)	0.40 (0.22–0.71)	0.62 (0.38–1.02)	0.81 (0.54–1.20)	
Model 4 (MSM-DAG)	1 (Ref)	1.08 (0.72–1.62)	0.47 (0.27–0.82)	0.65 (0.40–1.05)	0.82 (0.55–1.23)	
**Females**						
No. of participants	5,671	5,692	5,673	5,681	5,681	
Cases (person-years)	16 (38,592.03)	28 (70,305.85)	38 (82,307.07)	28 (84,549.44)	14 (52,137.62)	
Crude model	1 (Ref)	0.96 (0.52–1.78)	1.11 (0.62–1.99)	0.80 (0.43–1.47)	0.65 (0.32–1.32)	
Model 3 (DAG)	1 (Ref)	0.78 (0.40–1.53)	0.83 (0.41–1.69)	0.65 (0.32–1.28)	0.54 (0.25–1.16)	
Model 4 (MSM-DAG)	1 (Ref)	0.94 (0.50–1.76)	1.06 (0.59–1.93)	0.76 (0.41–1.43)	0.53 (0.35–1.11)	

Values are hazard ratios (95% confidence intervals).

^a^*P* for interaction was calculated by comparing models with and without interaction terms (sex stratification).

^b^A total of 782 colorectal cancer cases were identified, including 456 proximal colon cancer cases, 322 distal CRC (that is, distal colon and rectal cancer) cases, and 4 CRC cases with an unknown site.

^c^Incidence rate was calculated per 1,000 person-years.

^d^Crude model adjusted for none.

DAG refers to the directed acyclic graph used to identify potential confounders.

MSM refers to the Marginal Structural Model used to adjust the time-varying dietary exposure or variables that do not meet the PH assumption in the Cox regression models. Crude model adjusted for none.

Model 1 adjusted for age (years), and sex (male vs. female).

Model 2 adjusted for age (years), sex (male vs. female), race (white, non-Hispanic vs. black, non-Hispanic vs. Hispanic vs. others), physical activity (none vs. ≤ 1 h/week vs. ≥ 2 h/week), diabetes (no vs. yes), cigarette smoking (never vs. current vs. former), BMI (kg/m^2^), alcohol consumption (g/day), and energy from diet (kcal/day).

Model 3 adjusted the covariates in Model 2 using the Marginal Structural Model.

Model 4 adjusted for all variables, including age (years), sex (male vs. female), marital status (married vs. unmarried), race (white, non-Hispanic vs. black, non-Hispanic vs. Hispanic vs. others), education level (≤ some college vs. college graduate vs. postgraduate), physical activity (none vs. ≤ 1 h/week vs. ≥ 2 h/week), multivitamin use (no vs. yes), aspirin use (no vs. yes), diabetes (no vs. yes), cigarette smoking (never vs. current vs. former), pack-years (continuous), BMI (kg/m^2^), family history of colorectal cancer (no vs. yes vs. possibly), alcohol consumption (g/day), history of colorectal polyps (no vs. yes), history of colon comorbidities (no vs. yes), and energy from the diet (kcal/day). Model 5 adjusted all the covariates in Model 4 using the Marginal Structural Model.

### Dose-response analyses

**FIGURE 2:**
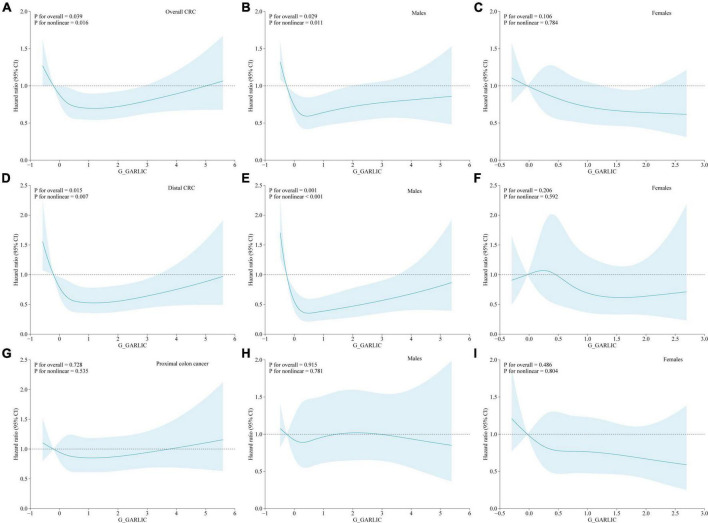
Non-linear dose-response associations between energy-adjusted dietary garlic consumption and colorectal cancer incidence based on a restricted cubic spline [**(A)** all CRC; **(B)** all CRC (males); **(C)** all CRC (females); **(D)** distal CRC; **(E)** distal CRC (males); **(F)** distal CRC (females); **(G)** proximal colon cancer; **(H)** proximal colon cancer (males); **(I)** proximal colon cancer (females)] in the whole study population. Hazard ratios and 95% confidence intervals of incident colorectal cancer were adjusted for age (years), sex (male vs. female), race (white, non-Hispanic vs. black, non-Hispanic vs. Hispanic vs. others), physical activity (none vs. ≤ 1 h/week. vs. ≥ 2 h/week.), diabetes (no vs. yes), cigarette smoking (never vs. current vs. former), BMI (kg/m^2^), alcohol consumption (g/day), and energy from diet (kcal/day), except the stratum group. The blue solid line represents the fitted non-linear trend, and the light blue area represents the corresponding 95% confidence interval.

We utilized restricted cubic spline plots to visualize the relationships between the dietary intake of garlic and the risk of CRC (overall CRC and its subsites: proximal colon cancer and distal CRC) across the full range of consumption levels. We observed a U-shaped curve between energy-adjusted garlic consumption and the risk of overall CRC (for both: Figure 2A: *P* for non-linearity = 0.016; for males: Figure 2B: *P* for non-linearity = 0.011), and the risk of distal CRC (for both: Figure 2D: *P* for non-linearity = 0.007; for males: Figure 2E: *P* for non-linearity < 0.001). However, no significant non-linear relationship was observed with the risk of proximal colon cancer or any location cancer type in females (Figures 2C, F–I: all *P* for non-linearity > 0.05).

### Additional analyses

The results of prespecified subgroups were displayed in Supplementary Table 3. Sex (overall CRC: *P* interaction = 0.008) and race (overall CRC: *P* interaction = 0.016; in males: *P* interaction = 0.01; and in females: *P* interaction = 0.003) were detected as significant and stable effect modifiers between energy-adjusted dietary garlic consumption concerning CRC incidence, respectively. To minimize the bias from small case numbers of subgroups, we categorized the exposure “garlic consumption” into tertiles and repeated the subgroup analyses (Supplementary Table 4). We found significant interaction terms among them (overall CRC: *P* interaction for sex = 0.026; *P* interaction for age = 0.031; *P* interaction for race = 0.025; and in males: *P* interaction for race = 0.02; and in females: *P* interaction for age = 0.03). Sensitivity analyses confirmed the robust inverse associations between energy-adjusted dietary garlic consumption and CRC risk (Supplementary Table 5).

## Discussion

In this large multicenter prospective cohort of the US general population, a significant association was observed between moderate dietary garlic consumption and a reduced risk of overall CRC incidence. This relationship exhibited a U-shaped dose-response manner. Notably, this association was more pronounced among men, Caucasians, and participants who consumed less alcohol in the whole population. Our study further demonstrated the robust protective effect of garlic on CRC incidence, a conclusion supported by a wide range of sensitivity analyses. To delve deeper into the potential correlations based on the anatomic subsites of CRC, we conducted exploratory analyses. These revealed a similar U-shaped inverse association with distal CRC risk but no such association was observed for proximal colon cancer risk.

Although a myriad of studies have explored the relationship between dietary garlic consumption and CRC incidence, the evidence is mainly from case-control studies and the results have been inconsistent. Some meta-analyses have reported an inverse association of dietary garlic with CRC incidence (16, 17), while others have found no such association (23, 24). Interestingly, when separately analyzed based on study type, the results showed that garlic was associated with reduced CRC risk in the case-control studies but the no such correlation in cohort studies (23). Meanwhile, a meta-analysis that only included cohort studies showed no association of colorectal cancer incidence with raw and cooked garlic or garlic supplements (24). This suggests that different study designs may have different effects on the results. Of course, it’s imperative to interpret the conclusions with caution because the evidence of an inverse association predominantly comes from case-control studies, which are potentially vulnerable to recall bias and selection bias. Moreover, the pooled studies exhibited significant heterogeneity. The prospective data on the impact of garlic on CRC incidence remains limited. Our study identified an inverse association between dietary garlic consumption and CRC incidence. This finding contrasts with recent prospective cohort studies, which reported no significant association between CRC incidence and either garlic intake or garlic supplement use (26, 27). The inconsistency might be attributed to variations in adjustments for potential confounders, sample sizes, and population heterogeneity. Furthermore, our study unveiled a U-shape dose-response relationship, a phenomenon yet to be explored by previous studies. Several factors might explain this observation, including the potential threshold effect of garlic’s protective compounds, the modulating effect of alcohol consumption, and the influence of dietary energy intake (38–40). Additionally, we noted a similar U-shaped inverse association with distal CRC risk, but no such association was evident for proximal colon cancer risk. This observation aligns with findings from the Iowa Women’s Health Study (IWHS) that involved 41,837 women (41). In our study, which involved 58,508 participants of both genders, we further corroborated the protective effect of dietary garlic intake on distal CRC. The heterogenous protective effect of dietary garlic intake on different CRC subsites offers valuable insights into the etiologic heterogeneity of CRC, potentially associated with the distinct molecular and microbial profiles of the proximal and distal colon (42, 43). Thus, gut microbiota may act as a potential mediator between diet and site-specific CRC risk (44, 45). This may be explained by the different regions of the gastrointestinal tract vary widely in terms of transit time, pH, exposure to oxygen, nutrient availability, mucosal surfaces, and interactions with the immune system, all of which affect microbial colonization (46). For example, there is a marked difference in the mucosal microbiota between patients who develop right- versus left-sided CRCs (43, 47–49), including in the presence of bacterial biofilms, defined as mucin layers with admixed bacteria on the luminal surface of the colonic epithelium, which can invade the mucus layer of the colon and may be pathogenic when they make direct contact with the mucosal epithelial cells. Invasive bacterial biofilms were found in 89% of right-sided CRCs but in only 12% of left-sided CRCs (50). In addition, evidence indicated that the short-chain fatty acids acetate, propionate, and butyrate function in the suppression of inflammation and cancer, whereas other microbial metabolites, such as secondary bile acids, promote carcinogenesis (46). For instance, one study showed that butyrate is a more important source of energy for the distal than the proximal colonic mucosa (51), which may be a relevant biological mechanism explaining the site-specific difference. Furthermore, garlic contains bioactive compounds, such as organosulfur compounds, that have been shown to have antimicrobial and anti-inflammatory properties. These compounds may influence the gut microbiota composition and function (52, 53), potentially leading to a protective effect against distal colorectal tumors. However, the findings related to the anatomical subsites of CRC require further experimental validation.

Interestingly, our finding highlighted a more pronounced reduction in CRC risk among men than women following a U-shaped dose-response manner. This disparity might be attributed to the variation in dietary and behavioral habits between genders, such as alcohol consumption and smoking patterns. Additionally, biological factors, especially hormonal differences might play a pivotal role (54). However, our result contrasts with a prospective cohort involving 579 men and 551 women of older US adults diagnosed with CRC (27). That study indicated that daily garlic consumption was associated with no significant correlation with CRC risk in men, whereas the association was nearly inverse in women. Although we cannot rule out the possibility of a chance finding, our results are derived from a study of 58,508 participants, providing a piece of more robust and powerful evidence. The specific mechanism to explain the sex disparities in tumorigenesis of CRC remains undetermined. In addition, racial and ethnic disparities in CRC risk are commonly documented in the literature which show a lower incidence and mortality of CRC among Caucasians (55, 56). These disparities may be attributed to differences in socioeconomic characteristics, dietary patterns, surveillance, and genetic and environmental factors. We also found the inverse association was more pronounced for the white race, but considering that over 90% of participants were non-Hispanic White, we should interpret the finding with caution, which needs to be further validated by future studies.

Our study, based on a large-scale, multi-center randomized trial with over 155,000 participants recruited from 10 screening centers, boasts an appropriate observation period, ensuring a substantial number of outcome events and minimizing the bias of reverse causality. However, certain limitations persist. First, using self-reported DQX to categorize food items may introduce non-differential misclassification bias. Second, despite thorough adjustments, we couldn’t eliminate all potential unmeasured confounders. Third, our one-time baseline assessment of food consumption might not diet habits change over time, though significant shifts in adult’s dietary habits over short periods are rare. Using only the baseline diet might weaken compared to cumulative averages. Fourth, our findings may not apply universally because generalized from the US population. Fifth, our study didn’t investigate garlic supplements due to data limitations.

## Conclusion

In US adults, moderate dietary garlic consumption shows a U-shaped dose-response association with a decreased risk of CRC. The protective association is particularly evident in distal CRC cases, but not in proximal colon cancer cases. Interestingly, the protective effect is more pronounced in men than in women, Caucasians, and among participants with lower alcohol consumption. Our findings underscore the importance of a healthy diet in mitigating the global burden of CRC.

## Data availability statement

The raw data supporting the conclusions of this article will be made available by the authors, without undue reservation.

## Ethics statement

Ethical approval was not required for the study involving humans in accordance with the local legislation and institutional requirements. The studies were conducted in accordance with the local legislation and institutional requirements. Written informed consent to participate in this study was not required from the participants in accordance with the national legislation and the institutional requirements.

## Author contributions

ZJ: Conceptualization, Data curation, Formal analysis, Investigation, Methodology, Project administration, Software, Supervision, Validation, Visualization, Writing – original draft, Writing – review & editing. HC: Conceptualization, Data curation, Formal analysis, Investigation, Methodology, Project administration, Software, Supervision, Validation, Visualization, Writing – original draft, Writing – review & editing. ML: Conceptualization, Data curation, Investigation, Methodology, Project administration, Supervision, Writing – review & editing. WW: Conceptualization, Data curation, Investigation, Methodology, Project administration, Supervision, Writing – review & editing. FL: Conceptualization, Data curation, Investigation, Methodology, Project administration, Supervision, Validation, Writing – original draft, Writing – review & editing. CF: Conceptualization, Data curation, Investigation, Methodology, Project administration, Supervision, Validation, Writing – original draft, Writing – review & editing.
