# Supplementary material for: Garlic consumption and colorectal cancer risk in US adults: a large prospective cohort study
Source: Front Nutr. 2023 Dec 6;10:1300330. doi: 10.3389/fnut.2023.1300330 (PMC10730668; doi:10.3389/fnut.2023.1300330)
Supplement: Supplementary file 1 [file Data_Sheet_1.docx]

***Supplementary Materials***

**Garlic consumption and colorectal cancer risk in US adults: a large prospective cohort study**

Zongze Jiang^1#^, Huilin Chen^2#^, Ming Li^1,3^, Wei Wang^1^, Feiwu Long^1*^, Chuanwen Fan^1,4*^

^1^Department of Gastrointestinal, Bariatric and Metabolic Surgery, Research Center for Nutrition, Metabolism and Food Safety, West China-PUMC C.C. Chen Institute of Health, West China School of Public Health and West China Fourth Hospital, Sichuan University, Chengdu, China

^2^Department of Immunology, Institute of Basic Medical Sciences, Chinese Academy of Medical Sciences; School of Basic Medicine, Peking Union Medical College, Beijing, China

^3^Department of Nutrition, Food Hygiene, and Toxicology, West China School of Public Health and West China Fourth Hospital, Sichuan University, Chengdu, China

^4^Department of Oncology and Department of Biomedical and Clinical Sciences, Linköping University, Linköping, Sweden

***Correspondence authors:**Feiwu Long (longfw1978@sina.com) and Chuanwen Fan (xuntian2005@163.com)

^#^ These authors contributed equally to this work.

***Keywords:*** garlic, colorectal cancer, adults, cohort study, epidemiology

***List of Supporting Information:***

**Supplementary Table 1.** Distribution of covariates with missing data before and after imputation.

**Supplementary Table 2.** The proportional hazard assumption of baseline covariates was verified by the Schoenfeld residuals in the Cox regression models.

**Supplementary Table 3.** Subgroup analyses on the associations between quintiles of energy-adjusted garlic consumption (g/day) and overall colorectal cancer incidence stratified by sex.

**Supplementary Table 4.** Subgroup analyses on the associations between tertiles of energy-adjusted garlic consumption (g/day) and overall colorectal cancer incidence.

**Supplementary Table 5.** Sensitivity analyses on the association between quintiles of energy-adjusted garlic consumption (g/day) and overall colorectal cancer incidence.

**Supplementary Figure 1.** The directed acyclic graph is constructed by the DAGityy (version 3.0; www.dagitty.net/). The variable below the green oval with a symbol “▲” (i.e., garlic consumption) represents the exposure variable; the variable below the blue oval with a symbol “┃” (i.e., colorectal cancer) represents the outcome variable; the variables below the pink oval represent the ancestor of exposure and outcome; and the variables below the green oval represent the ancestor of exposure. BMI, body mass index.


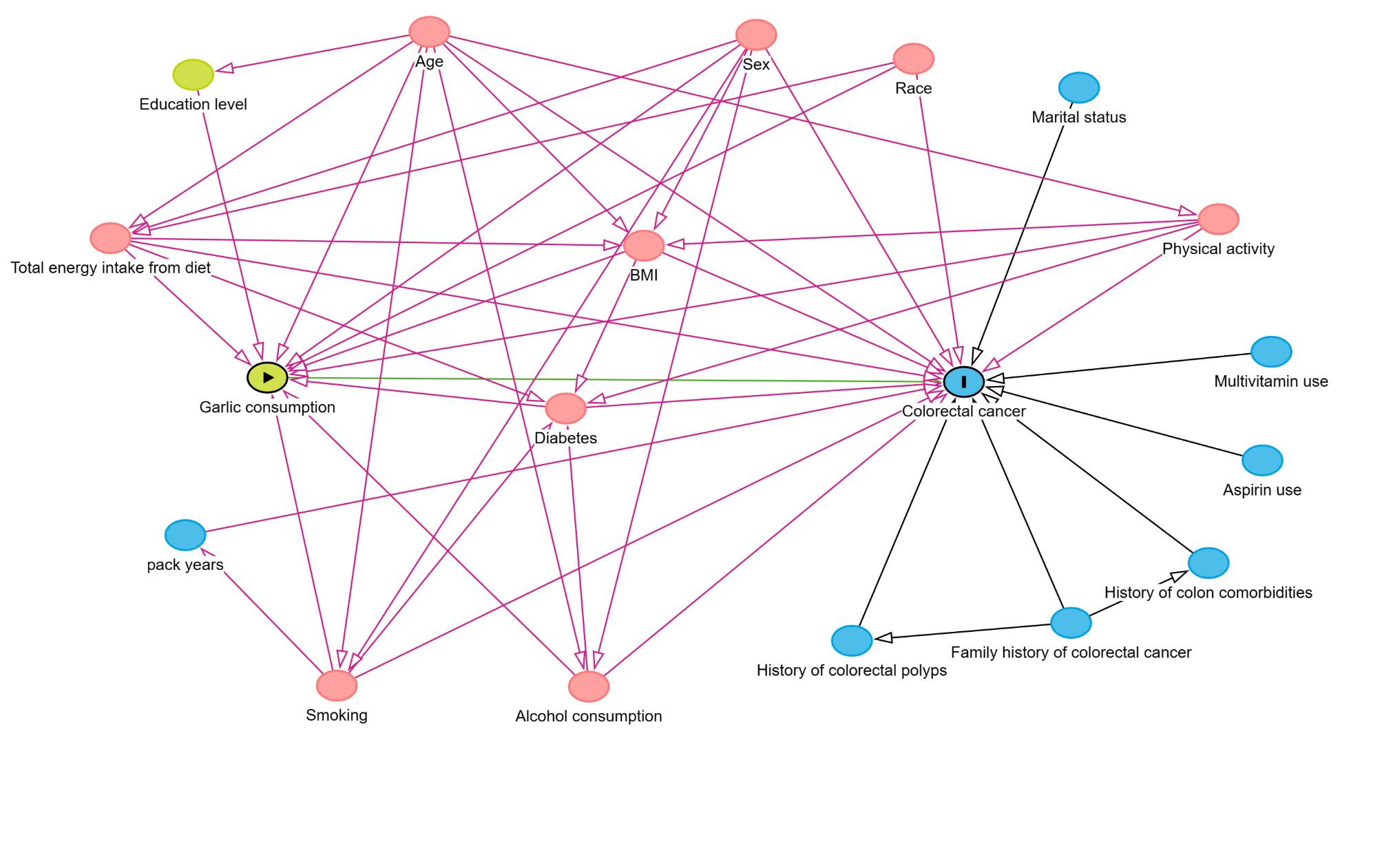


| Supplementary Table 1. Distribution of covariates with missing data before and after imputation. | | | |  |
| --- | --- | --- | --- | --- |
| Covariates | Before imputation | After imputation | Number of missing (%) |  |
| Race (%) |  |  | 18 (0.03) |  |
| White, Non-Hispanic | 53131 (90.84) | 53149 (90.84) |  |  |
| Black, Non-Hispanic | 2245 (3.84) | 2245 (3.84) |  |  |
| Hispanic | 887 (1.52) | 887 (1.52) |  |  |
| Others | 2227 (3.81) | 2227 (3.81) |  |  |
| Education level (%) |  |  | 49 (0.08) |  |
| College below | 37422 (64.01) | 37468 (64.04) |  |  |
| College graduate | 10205 (17.46) | 10206 (17.44) |  |  |
| Postgraduate | 10832 (18.53) | 10834 (18.52) |  |  |
| Marital status (%) |  |  | 43 (0.07) |  |
| Married | 45804 (78.34) | 45847 (78.36) |  |  |
| Unmarried | 12661 (21.66) | 12661 (21.64) |  |  |
| Smoking status (%) |  |  | 5 (0.01) |  |
| Never | 27407 (46.85) | 27409 (46.85) |  |  |
| Current | 5745 (9.82) | 5745 (9.82) |  |  |
| Former | 25351 (43.33) | 25354 (43.33) |  |  |
| Pack years | 18.48±27.26 | 18.66±27.21 | 610 (1.04) |  |
| Family history of colorectal cancer (%) |  |  | 422 (0.72) |  |
| No | 50437 (86.83) | 50859 (86.93) |  |  |
| Yes | 6074 (10.46) | 6074 (10.38) |  |  |
| Possibly | 1575 (2.71) | 1575 (2.69) |  |  |
| BMI (kg/m^2^) | 27.28±4.80 | 27.28±4.78 | 569 (0.97) |  |
| Aspirin use (%) |  |  | 194 (0.33) |  |
| No | 30765 (52.76) | 30875 (52.77) |  |  |
| Yes | 27549 (47.24) | 27633 (47.23) |  |  |
| History of colon comorbidities (%) |  |  | 346 (0.59) |  |
| No | 57355 (98.61) | 57701 (98.62) |  |  |
| Yes | 807 (1.39) | 807 (1.38) |  |  |
| History of polyps (%) |  |  | 207 (0.35) |  |
| No | 54182 (92.93) | 54389 (92.96) |  |  |
| Yes | 4119 (7.07) | 4119 (7.04) |  |  |
| History of diabetes (%) |  |  | 159 (0.27) |  |
| No | 54167 (92.83) | 54326 (92.85) |  |  |
| Yes | 4182 (7.17) | 4182 (7.15) |  |  |
| Physical activity (%) |  |  | 206 (0.35) |  |
| None | 8916 (15.29) | 8924 (15.25) |  |  |
| <=1 hour/wk. | 17298 (29.67) | 17317 (29.60) |  |  |
| >=2 hours/wk. | 32088 (55.04) | 32267 (55.15) |  |  |
| Values are mean ± standard deviation or counts (percentage) as indicated. "Others" refers to Hispanic, Asian, Pacific Islander, or American Indian; BMI, body mass index; | | | |  |
|  |  |  |  |  |

| Supplementary Table 2. The proportional hazard assumption of baseline covariates was verified by the Schoenfeld residuals in the Cox regression models. | | | | | | | | | | | | | | |  |  |  |  |  |
| --- | --- | --- | --- | --- | --- | --- | --- | --- | --- | --- | --- | --- | --- | --- | --- | --- | --- | --- | --- |
|  | | PH assumption | | | | | | | | | | | | |  |  |  |  |  |
|  | | Colorectal cancer | |  | | | Proximal colon cancer | | | | Distal colorectal cancer | | | |  |  |  |  |  |
| Variables | | Garlic (quintiles) | | Garlic (continuous) | | | Garlic (quintiles) | Garlic (continuous) | | | Garlic (quintiles) | | | Garlic (continuous) |  |  |  |  |  |
| Model (DAGs) | |  | |  | | |  |  | | |  | | |  |  |  |  |  |  |
| G_GARLIC | | 0.226 | | 0.952 | | | 0.41 | 0.66 | | | 0.265 | | | 0.724 |  |  |  |  |  |
| Age_dqx | | 0.282 | | 0.285 | | | 0.21 | 0.21 | | | 0.228 | | | 0.229 |  |  |  |  |  |
| sex | | **0.023** | | **0.023** | | | 0.67 | 0.67 | | | **0.034** | | | **0.034** |  |  |  |  |  |
| physical_activity_now | | 0.641 | | 0.643 | | | 0.94 | 0.94 | | | 0.69 | | | 0.69 |  |  |  |  |  |
| Alcohol_consumption | | 0.158 | | 0.158 | | | 0.7 | 0.7 | | | 0.094 | | | 0.095 |  |  |  |  |  |
| DT_KCAL | | 0.287 | | 0.294 | | | 0.42 | 0.42 | | | 0.39 | | | 0.401 |  |  |  |  |  |
| Race | | 0.08 | | 0.079 | | | 0.16 | 0.16 | | | 0.211 | | | 0.21 |  |  |  |  |  |
| cig_stat | | 0.921 | | 0.923 | | | 0.7 | 0.7 | | | 0.923 | | | 0.927 |  |  |  |  |  |
| bmi_curr | | 0.091 | | 0.091 | | | 0.33 | 0.33 | | | 0.15 | | | 0.151 |  |  |  |  |  |
| diabetes_f | | 0.596 | | 0.596 | | | 0.12 | 0.12 | | | 0.882 | | | 0.882 |  |  |  |  |  |
| GLOBAL | | 0.057 | | 0.121 | | | 0.39 | 0.48 | | | 0.164 | | | 0.265 |  |  |  |  |  |
|  | |  | |  | | |  |  | | |  | | |  |  |  |  |  |  |
| Model (Sex stratification) | |  | |  | | |  |  | | |  | | |  |  |  |  |  |  |
| G_GARLIC | | 0.246 | | 0.823 | | | 0.4 | 0.64 | | | 0.29 | | | 0.82 |  |  |  |  |  |
| Age_dqx | | 0.261 | | 0.264 | | | 0.22 | 0.22 | | | 0.21 | | | 0.21 |  |  |  |  |  |
| physical_activity_now | | 0.594 | | 0.596 | | | 0.93 | 0.93 | | | 0.65 | | | 0.65 |  |  |  |  |  |
| Alcohol_consumption | | 0.354 | | 0.354 | | | 0.62 | 0.63 | | | 0.21 | | | 0.21 |  |  |  |  |  |
| DT_KCAL | | 0.784 | | 0.803 | | | 0.48 | 0.49 | | | 0.89 | | | 0.91 |  |  |  |  |  |
| Race | | 0.087 | | 0.086 | | | 0.16 | 0.16 | | | 0.23 | | | 0.23 |  |  |  |  |  |
| cig_stat | | 0.556 | | 0.558 | | | 0.63 | 0.62 | | | 0.59 | | | 0.6 |  |  |  |  |  |
| bmi_curr | | 0.08 | | 0.08 | | | 0.32 | 0.33 | | | 0.13 | | | 0.13 |  |  |  |  |  |
| diabetes_f | | 0.685 | | 0.685 | | | 0.13 | 0.13 | | | 0.81 | | | 0.81 |  |  |  |  |  |
| GLOBAL | | 0.164 | | 0.352 | | | 0.33 | 0.41 | | | 0.35 | | | 0.56 |  |  |  |  |  |
|  | |  | |  | | |  |  | | |  | | |  |  |  |  |  |  |
| Model (all covariates) | |  | |  | | |  |  | | |  | | |  |  |  |  |  |  |
| G_GARLIC | | 0.2274 | | 0.9444 | | | 0.415 | 0.662 | | | 0.267 | | | 0.732 |  |  |  |  |  |
| Age_dqx | | 0.2782 | | 0.2807 | | | 0.21 | 0.208 | | | 0.223 | | | 0.223 |  |  |  |  |  |
| sex | | **0.0252** | | **0.0251** | | | 0.664 | 0.665 | | | **0.037** | | | **0.037** |  |  |  |  |  |
| physical_activity_now | | 0.6493 | | 0.6503 | | | 0.941 | 0.941 | | | 0.699 | | | 0.7 |  |  |  |  |  |
| Alcohol_consumption | | 0.171 | | 0.1711 | | | 0.695 | 0.697 | | | 0.107 | | | 0.108 |  |  |  |  |  |
| multivatimin_use | | 0.069 | | 0.0688 | | | 0.318 | 0.319 | | | 0.162 | | | 0.161 |  |  |  |  |  |
| DT_KCAL | | 0.2983 | | 0.3062 | | | 0.413 | 0.417 | | | 0.407 | | | 0.418 |  |  |  |  |  |
| Race | | 0.0793 | | 0.0786 | | | 0.163 | 0.164 | | | 0.208 | | | 0.207 |  |  |  |  |  |
| educat | | 0.2636 | | 0.264 | | | 0.686 | 0.687 | | | 0.939 | | | 0.94 |  |  |  |  |  |
| marital | | 0.1733 | | 0.1734 | | | 0.094 | 0.094 | | | 0.329 | | | 0.33 |  |  |  |  |  |
| cig_stat | | 0.9107 | | 0.9128 | | | 0.711 | 0.709 | | | 0.906 | | | 0.91 |  |  |  |  |  |
| pack_years | | 0.4021 | | 0.397 | | | 0.306 | 0.304 | | | 0.733 | | | 0.74 |  |  |  |  |  |
| family_history_colorectal_cancer | | 0.1236 | | 0.1239 | | | 0.162 | 0.162 | | | 0.45 | | | 0.451 |  |  |  |  |  |
| asp | | **0.0084** | | **0.0082** | | | 0.063 | 0.063 | | | **0.019** | | | **0.018** |  |  |  |  |  |
| colon_comorbidity | | 0.0705 | | 0.0707 | | | 0.877 | 0.878 | | | 0.029 | | | 0.029 |  |  |  |  |  |
| bmi_curr | | 0.0957 | | 0.0965 | | | 0.338 | 0.34 | | | 0.156 | | | 0.156 |  |  |  |  |  |
| diabetes_f | | 0.5965 | | 0.5965 | | | 0.118 | 0.118 | | | 0.872 | | | 0.871 |  |  |  |  |  |
| polyps_f | | 0.7409 | | 0.7458 | | | 0.111 | 0.109 | | | 0.346 | | | 0.346 |  |  |  |  |  |
| GLOBAL | | **0.0046** | | **0.0078** | | | 0.186 | 0.212 | | | 0.088 | | | 0.128 |  |  |  |  |  |
|  | |  | |  | | |  |  | | |  | | |  |  |  |  |  |  |
|  | |  | |  | | |  |  | | |  | | |  |  |  |  |  |  |
|  | |  | |  | | |  |  | | |  | | |  |  |  |  |  |  |
| **Supplementary Table 3**. Subgroup analyses on the associations between quintiles of energy-adjusted garlic consumption (g/day) and overall colorectal cancer incidence. | | | | | | | | | | | | | | | | | | |  |
|  | Quintiles of energy-adjusted garlic consumption (g/day), HR (95 % CI), *p*-value | | | | | | | | | | | | | | | | | |  |
| Subgroup variables | Quintile 1 | | Quintile 2 | | *p* | Quintile 3 | | | *p* | Quintile 4 | | *p* | Quintile 5 | | | *p* | *P trend ^a^* | *P interaction ^b^* |  |
| **Overall** |  | |  | |  |  | | |  |  | |  |  | | |  |  |  |  |
| Age (years) |  | |  | |  |  | | |  |  | |  |  | | |  |  | 0.052 |  |
| < Median | ref | | 0.88(0.58,1.35) | | 0.563 | 0.72 (0.46,1.14) | | | 0.163 | 0.66 (0.43,1.02) | | 0.064 | 0.65(0.43,0.99) | | | 0.042 | 0.052 |  |  |
| >=Median | ref | | 0.95(0.72,1.25) | | 0.702 | 0.69(0.50,0.94) | | | 0.020 | 0.90(0.67,1.20) | | 0.461 | 0.94(0.72,1.24) | | | 0.676 | 0.822 |  |  |
| Sex |  | |  | |  |  | | |  |  | |  |  | | |  |  | **0.008** |  |
| Male | ref | | 0.94(0.70,1.25) | | 0.661 | 0.57(0.40,0.81) | | | 0.002 | 0.88(0.65,1.20) | | 0.417 | 0.90(0.69,1.17) | | | 0.416 | 0.887 |  |  |
| Female | ref | | 0.89(0.59,1.34) | | 0.583 | 0.77(0.49,1.19) | | | 0.235 | 0.74(0.49,1.13) | | 0.160 | 0.71(0.45,1.11) | | | 0.138 | 0.16 |  |  |
| Race |  | |  | |  |  | | |  |  | |  |  | | |  |  | **0.016** |  |
| White, Non-Hispanic | ref | | 0.91(0.72,1.16) | | 0.445 | 0.71(0.54,0.93) | | | 0.012 | 0.83(0.64,1.07) | | 0.149 | 0.84(0.66,1.08) | | | 0.170 | 0.42 |  |  |
| Black, Non-Hispanic | ref | | 0.99(0.39,2.52) | | 0.981 | 0.56(0.19,1.68) | | | 0.301 | 0.46(0.14,1.50) | | 0.198 | 0.51(0.20,1.31) | | | 0.161 | 0.138 |  |  |
| Others | ref | | 0.83(0.18,3.91) | | 0.812 | 0.56 (0.11,2.76) | | | 0.477 | 1.08(0.31,3.78) | | 0.908 | 1.25(0.41,3.86) | | | 0.696 | 0.281 |  |  |
| BMI (kg/m2) |  | |  | |  |  | | |  |  | |  |  | | |  |  | 0.079 |  |
| <25 | ref | | 0.98(0.64,1.50) | | 0.924 | 0.61(0.37,0.99) | | | 0.047 | 0.67 (0.42,1.06) | | 0.089 | 0.81(0.52,1.26) | | | 0.351 | 0.514 |  |  |
| >=25 | ref | | 0.89(0.67,1.17) | | 0.394 | 0.74(0.54,1.00) | | | 0.049 | 0.88 (0.67,1.17) | | 0.385 | 0.84(0.64,1.10) | | | 0.203 | 0.476 |  |  |
| Smoking status |  | |  | |  |  | | |  |  | |  |  | | |  |  | 0.085 |  |
| Current or former | ref | | 1.06(0.78,1.44) | | 0.705 | 0.73(0.52,1.02) | | | 0.065 | 0.78(0.57,1.08) | | 0.133 | 0.85(0.63,1.14) | | | 0.275 | 0.286 |  |  |
| Never | ref | | 0.77(0.54,1.10) | | 0.146 | 0.66(0.44,0.98) | | | 0.038 | 0.86(0.59,1.24) | | 0.406 | 0.83 (0.58,1.20) | | | 0.321 | 0.925 |  |  |
| Alcohol consumption (g/day) ^c^ |  | |  | |  |  | | |  |  | |  |  | | |  |  | **0.035** |  |
| No, light or moderate | ref | | 0.91(0.71,1.18) | | 0.482 | 0.66(0.49,0.88) | | | 0.004 | 0.82(0.63,1.07) | | 0.144 | 0.78(0.60,1.01) | | | 0.056 | 0.182 |  |  |
| Heavy | ref | | 0.81(0.43,1.52) | | 0.511 | 0.87(0.46,1.62) | | | 0.654 | 0.74 (0.42,1.30) | | 0.294 | 1.06(0.66,1.70) | | | 0.823 | 0.512 |  |  |
|  |  | |  | |  |  | | |  |  | |  |  | | |  |  |  |  |
| **Males** |  | |  | |  |  | | |  |  | |  |  | | |  |  |  |  |
| Age (years) |  | |  | |  |  | | |  |  | |  |  | | |  |  | 0.08 |  |
| < Median | ref | | 0.89(0.47, 1.68) | | 0.72 | 0.87(0.46, 1.64) | | | 0.66 | 1.02(0.57, 1.82) | | 0.95 | 0.73(0.40, 1.35) | | | 0.32 | 0.34 |  |  |
| >=Median | ref | | 0.63(0.43, 0.93) | | 0.02 | 0.49(0.33, 0.74) | | | <0.001 | 0.7(0.48, 1.02) | | 0.06 | 0.72(0.50, 1.04) | | | 0.08 | 0.94 |  |  |
| Race |  | |  | |  |  | | |  |  | |  |  | | |  |  | **0.01** |  |
| White, Non-Hispanic | ref | | 0.74(0.53,1.04) | | 0.09 | 0.6(0.42,0.86) | | | 0.01 | 0.86(0.62,1.19) | | 0.36 | 0.79(0.57,1.11) | | | 0.17 | 0.91 |  |  |
| Black, Non-Hispanic | ref | | 0.46(0.10, 2.11) | | 0.32 | 0.52(0.12, 2.27) | | | 0.39 | 0.32(0.06, 1.65) | | 0.17 | 0.22(0.05, 1.01) | | | 0.05 | 0.07 |  |  |
| Others | ref | | 0.3(0.05, 1.93) | | 0.21 | 0.21(0.04,1.18) | | | 0.08 | 0.41(0.10,1.67) | | 0.21 | 0.45(0.12,1.64) | | | 0.22 | 0.85 |  |  |
| BMI (kg/m2) |  | |  | |  |  | | |  |  | |  |  | | |  |  | 0.23 |  |
| <25 | ref | | 0.84(0.43, 1.64) | | 0.61 | 0.68(0.34, 1.38) | | | 0.29 | 0.78(0.40, 1.51) | | 0.46 | 0.77(0.40, 1.49) | | | 0.44 | 0.67 |  |  |
| >=25 | ref | | 0.65(0.45, 0.95) | | 0.03 | 0.55(0.37, 0.81) | | | 0.003 | 0.79(0.55, 1.12) | | 0.19 | 0.71(0.50, 1.01) | | | 0.06 | 0.67 |  |  |
| Smoking status |  | |  | |  |  | | |  |  | |  |  | | |  |  | 0.1 |  |
| Current or former | ref | | 0.43(0.24, 0.77) | | 0.005 | 0.45(0.25, 0.80) | | | 0.01 | 0.71(0.42, 1.21) | | 0.21 | 0.63(0.36, 1.10) | | | 0.10 | 0.95 |  |  |
| Never | ref | | 0.87(0.58, 1.30) | | 0.50 | 0.64(0.42, 0.98) | | | 0.04 | 0.82(0.55, 1.21) | | 0.31 | 0.77(0.53, 1.13) | | | 0.19 | 0.47 |  |  |
| Alcohol consumption (g/day) ^c^ |  | |  | |  |  | | |  |  | |  |  | | |  |  | **0.004** |  |
| No, light or moderate | ref | | 0.76(0.53, 1.11) | | 0.15 | 0.61(0.42, 0.90) | | | 0.01 | 0.9(0.63, 1.29) | | 0.57 | 0.69(0.47, 1.00) | | | 0.05 | 0.33 |  |  |
| Heavy | ref | | 0.45(0.20, 0.99) | | 0.05 | 0.48(0.22, 1.07) | | | 0.07 | 0.46(0.23, 0.92) | | 0.03 | 0.82(0.47, 1.44) | | | 0.49 | 0.56 |  |  |
|  |  | |  | |  |  | | |  |  | |  |  | | |  |  |  |  |
| **Females** |  | |  | |  |  | | |  |  | |  |  | | |  |  |  |  |
| Age (years) |  | |  | |  |  | | |  |  | |  |  | | |  |  | **< 0.001** |  |
| < Median | ref | | 0.72(0.39, 1.32) | | 0.29 | 0.9(0.50, 1.62) | | | 0.74 | 0.42(0.22, 0.80) | | 0.01 | 0.49(0.26, 0.91) | | | 0.02 | 0.01 |  |  |
| >=Median | ref | | 1.11(0.71, 1.74) | | 0.65 | 0.86(0.53, 1.40) | | | 0.54 | 1.25(0.82, 1.91) | | 0.31 | 0.85(0.53, 1.37) | | | 0.51 | 0.56 |  |  |
| Race |  | |  | |  |  | | |  |  | |  |  | | |  |  | **0.03** |  |
| White, Non-Hispanic | ref | | 0.92(0.64, 1.34) | | 0.68 | 0.85(0.57,1.25) | | | 0.40 | 0.82(0.57, 1.19) | | 0.30 | 0.64(0.42, 0.96) | | | 0.03 | 0.03 |  |  |
| Black, Non-Hispanic | ref | | 1.81(0.40, 8.10) | | 0.44 | 2.17(0.45, 10.52) | | | 0.34 | 1.38(0.30, 6.40) | | 0.68 | 1.4(0.38, 5.19) | | | 0.61 | 0.84 |  |  |
| Others | ref | | 0.99(0.00, 2.03e+31) | | 1.00 | 383.99(0.00, 6.93e+25) | | | 0.83 | 957.95(0.00, 1.67e+26) | | 0.80 | 540.37 (0.00,9.44e+25) | | | 0.82 | 0.27 |  |  |
| BMI (kg/m2) |  | |  | |  |  | | |  |  | |  |  | | |  |  | 0.28 |  |
| <25 | ref | | 1.07(0.59, 1.95) | | 0.82 | 0.85(0.45, 1.59) | | | 0.61 | 0.96(0.54, 1.73) | | 0.90 | 0.75(0.40, 1.40) | | | 0.36 | 0.28 |  |  |
| >=25 | ref | | 0.92(0.58, 1.44) | | 0.71 | 0.94(0.59, 1.50) | | | 0.79 | 0.85(0.55, 1.32) | | 0.47 | 0.67(0.42, 1.08) | | | 0.10 | 0.08 |  |  |
| Smoking status |  | |  | |  |  | | |  |  | |  |  | | |  |  |  |  |
| Current or former | ref | | 1.01(0.63, 1.62) | | 0.96 | 0.82(0.49, 1.37) | | | 0.44 | 1.12(0.71, 1.76) | | 0.62 | 0.66(0.38, 1.13) | | | 0.13 | 0.19 |  |  |
| Never | ref | | 0.9(0.52, 1.57) | | 0.72 | 0.96(0.55, 1.67) | | | 0.88 | 0.68(0.39, 1.16) | | 0.16 | 0.72(0.42, 1.23) | | | 0.23 | 0.13 |  |  |
| Alcohol consumption (g/day) ^c^ |  | |  | |  |  | | |  |  | |  |  | | |  |  | 0.11 |  |
| No, light or moderate | ref | | 0.88(0.60, 1.29) | | 0.52 | 0.81(0.55, 1.21) | | | 0.31 | 0.84(0.58, 1.22) | | 0.35 | 0.62(0.41, 0.94) | | | 0.02 | 0.03 |  |  |
| Heavy | ref | | 1.83(0.57, 5.85) | | 0.31 | 1.67(0.52, 5.36) | | | 0.39 | 1.33(0.45, 3.94) | | 0.60 | 1.43(0.48, 4.26) | | | 0.52 | 0.99 |  |  |
| ^a^ Trend test was performed using median value of each diet score quintile as a continuous variable. ^b^ *P* value for interaction was estimated using the likelihood ratio test comparing the model with and without the interaction term of the energy-adjusted dietary garlic consumption and the respective stratification variable.  ^c^ Light, moderate, and heavy alcohol consumption are defined as <=6 g/day, > 6–28 g/day for male and > 6–14 g/day for female, and > 28 g/day for male and > 14 g/day for female, respectively. Hazard ratios and 95% confidence intervals of incident colorectal cancer were adjusted for age (<median vs. >=median), sex (male vs. female), race (white, non-Hispanic vs. black, non-Hispanic vs. others), physical activity (none vs. <=1 hour/week vs. >=2 hours/week), diabetes (no vs. yes), cigarette smoking (never vs. current/former), BMI (<25 vs. >=25 kg/m2), alcohol consumption (no/light/moderate vs. heavy), and energy from diet (kcal/day), except the stratification factor. | | | | | | | | | | | | | | | | | | |  |
|  |  |  |  |  |  |  |  |  |  |  |  |  |  |  |  |  |  |  |  |
|  |  |  |  |  |  |  |  |  |  |  |  |  |  |  |  |  |  |  |  |
|  |  |  |  |  |  |  |  |  |  |  |  |  |  |  |  |  |  |  |  |
|  |  |  |  |  |  |  |  |  |  |  |  |  |  |  |  |  |  |  |  |
|  |  |  |  |  |  |  |  |  |  |  |  |  |  |  |  |  |  |  |  |
|  |  |  |  |  |  |  |  |  |  |  |  |  |  |  |  |  |  |  |  |
|  |  |  |  |  |  |  |  |  |  |  |  |  |  |  |  |  |  |  |  |
|  |  | |  | |  |  | | |  |  | |  |  | | |  |  |  |  |

| **Supplementary Table 4**. Subgroup analyses on the associations between tertiles of energy-adjusted garlic consumption (g/day) and overall colorectal cancer incidence. | | | | | | | | | | | | |  |  |  |  |  |
| --- | --- | --- | --- | --- | --- | --- | --- | --- | --- | --- | --- | --- | --- | --- | --- | --- | --- |
|  | Tertiles of energy-adjusted garlic consumption (g/day), HR (95%CI), *p*-value | | | | | | | | | | | |  |  |  |  |  |
| Subgroup variables | Tertile 1 | Tertile 2 | | | *p* | Tertile 3 | | *p* | *P trend ^a^* | | *P interaction ^b^* | |  |  |  |  |  |
| **Overall CRC** |  |  | | |  |  | |  |  | |  | |  |  |  |  |  |
| Age (years) |  |  | | |  |  | |  |  | | **0.031** | |  |  |  |  |  |
| < Median | ref | 0.94(0.68,1.32) | | | 0.74 | 0.69(0.50,0.97) | | 0.03 | 0.02 | |  | |  |  |  |  |  |
| >=Median | ref | 0.86(0.69,1.07) | | | 0.18 | 0.97(0.79,1.20) | | 0.80 | 0.85 | |  | |  |  |  |  |  |
| Sex |  |  | | |  |  | |  |  | | **0.026** | |  |  |  |  |  |
| Male | ref | 0.78(0.61,1.01) | | | 0.06 | 0.93(0.75,1.16) | | 0.52 | 0.85 | |  | |  |  |  |  |  |
| Female | ref | 0.96(0.72,1.30) | | | 0.81 | 0.8(0.59,1.09) | | 0.16 | 0.11 | |  | |  |  |  |  |  |
| Race |  |  | | |  |  | |  |  | | **0.025** | |  |  |  |  |  |
| White, Non-Hispanic | ref | 0.9(0.74,1.09) | | | 0.26 | 0.9(0.74,1.08) | | 0.25 | 0.37 | |  | |  |  |  |  |  |
| Black, Non-Hispanic | ref | 0.76(0.35,1.67) | | | 0.50 | 0.47(0.21,1.02) | | 0.06 | 0.06 | |  | |  |  |  |  |  |
| Others | ref | 0.78(0.25,2.47) | | | 0.67 | 1.23(0.49,3.07) | | 0.66 | 0.36 | |  | |  |  |  |  |  |
| BMI (kg/m2) |  |  | | |  |  | |  |  | | 0.593 | |  |  |  |  |  |
| <25 | ref | 0.85(0.61,1.19) | | | 0.34 | 0.82(0.59,1.14) | | 0.24 | 0.34 | |  | |  |  |  |  |  |
| >=25 | ref | 0.9(0.72,1.12) | | | 0.35 | 0.9(0.73,1.11) | | 0.31 | 0.42 | |  | |  |  |  |  |  |
| Smoking status |  |  | | |  |  | |  |  | | 0.339 | |  |  |  |  |  |
| Current or former | ref | 0.83(0.65,1.07) | | | 0.14 | 0.81(0.64,1.02) | | 0.07 | 0.14 | |  | |  |  |  |  |  |
| Never | ref | 0.96(0.72,1.27) | | | 0.77 | 0.99(0.75,1.31) | | 0.93 | 1 | |  | |  |  |  |  |  |
| Alcohol consumption (g/day) ^c^ |  |  | | |  |  | |  |  | | 0.134 | |  |  |  |  |  |
| No, light or moderate | ref | 0.88(0.72,1.07) | | | 0.20 | 0.84(0.69,1.02) | | 0.08 | 0.13 | |  | |  |  |  |  |  |
| Heavy | ref | 0.84(0.51,1.40) | | | 0.51 | 1.04(0.70,1.56) | | 0.85 | 0.63 | |  | |  |  |  |  |  |
|  |  |  | | |  |  | |  |  | |  | |  |  |  |  |  |
| **Males** |  |  | | |  |  | |  |  | |  | |  |  |  |  |  |
| Age (years) |  |  | | |  |  | |  |  | | 0.25 | |  |  |  |  |  |
| < Median | ref | 0.88(0.55,1.40) | | | 0.59 | 0.84(0.54,1.31) | | 0.45 | 0.54 | |  | |  |  |  |  |  |
| >=Median | ref | 0.69(0.52,0.91) | | | 0.01 | 0.87(0.67,1.14) | | 0.33 | 0.98 | |  | |  |  |  |  |  |
| Race |  |  | | |  |  | |  |  | | **0.02** | |  |  |  |  |  |
| White, Non-Hispanic | ref | 0.73(0.56,0.94) | | | 0.01 | 0.9(0.71,1.15) | | 0.42 | 0.91 | |  | |  |  |  |  |  |
| Black, Non-Hispanic | ref | 0.91(0.32, 2.57) | | | 0.86 | 0.31(0.09, 1.05) | | 0.06 | 0.04 | |  | |  |  |  |  |  |
| Others | ref | 0.74(0.20,2.71) | | | 0.65 | 1.01(0.33,3.12) | | 0.99 | 0.62 | |  | |  |  |  |  |  |
| BMI (kg/m2) |  |  | | |  |  | |  |  | | 0.36 | |  |  |  |  |  |
| <25 | ref | 0.77(0.48,1.25) | | | 0.29 | 0.79(0.49,1.27) | | 0.33 |  | |  | |  |  |  |  |  |
| >=25 | ref | 0.72(0.54,0.95) | | | 0.02 | 0.88(0.68,1.15) | | 0.36 |  | |  | |  |  |  |  |  |
| Smoking status |  |  | | |  |  | |  |  | | 0.42 | |  |  |  |  |  |
| Current or former | ref | 0.77(0.57,1.03) | | | 0.08 | 0.83(0.62,1.10) | | 0.19 | 0.49 | |  | |  |  |  |  |  |
| Never | ref | 0.66(0.44,1.01) | | | 0.05 | 0.94(0.63,1.39) | | 0.75 | 0.66 | |  | |  |  |  |  |  |
| Alcohol consumption (g/day) ^c^ |  |  | | |  |  | |  |  | | 0.12 | |  |  |  |  |  |
| No, light or moderate | ref | 0.77(0.59,1.00) | | | 0.05 | 0.85(0.65,1.11) | | 0.23 | 0.63 | |  | |  |  |  |  |  |
| Heavy | ref | 0.53(0.28,1.01) | | | 0.05 | 0.9(0.56,1.44) | | 0.66 | 0.77 | |  | |  |  |  |  |  |
|  |  |  | | |  |  | |  |  | |  | |  |  |  |  |  |
| **Females** |  |  | | |  |  | |  |  | |  | |  |  |  |  |  |
| Age (years) |  |  | | |  |  | |  |  | | **0.03** | |  |  |  |  |  |
| < Median | ref | 1(0.64, 1.57) | | | 0.99 | 0.56(0.34, 0.92) | | **0.02** | **0.01** | |  | |  |  |  |  |  |
| >=Median | ref | 0.89(0.64,1.25) | | | 0.52 | 0.95(0.68,1.33) | | 0.77 | 0.9 | |  | |  |  |  |  |  |
| Race |  |  | | |  |  | |  |  | | 0.07 | |  |  |  |  |  |
| White, Non-Hispanic | ref | 0.9(0.68,1.19) | | | 0.47 | 0.76(0.57,1.02) | | 0.07 | 0.07 | |  | |  |  |  |  |  |
| Black, Non-Hispanic | ref | 1.63(0.51,5.19) | | | 0.41 | 0.96(0.33,2.77) | | 0.93 | 0.71 | |  | |  |  |  |  |  |
| Others | ref | 494.51(0.00,1.73e+19) | | | 0.75 | 634.07(0.00,2.17e+19) | | 0.74 | 0.29 | |  | |  |  |  |  |  |
| BMI (kg/m2) |  |  | | |  |  | |  |  | | 0.59 | |  |  |  |  |  |
| <25 | ref | 0.81(0.52, 1.25) | | | 0.34 | 0.76(0.49, 1.19) | | 0.23 | 0.31 | |  | |  |  |  |  |  |
| >=25 | ref | 1.03(0.73,1.45) | | | 0.87 | 0.81(0.57,1.16) | | 0.25 | 0.19 | |  | |  |  |  |  |  |
| Smoking status |  |  | | |  |  | |  |  | | 0.67 | |  |  |  |  |  |
| Current or former | ref | 0.86(0.57,1.29) | | | 0.46 | 0.73(0.49,1.10) | | 0.13 | 0.15 | |  | |  |  |  |  |  |
| Never | ref | 1(0.70, 1.44) | | | 0.98 | 0.86(0.59, 1.26) | | 0.44 | 0.4 | |  | |  |  |  |  |  |
| Alcohol consumption (g/day) ^c^ |  |  | | |  |  | |  |  | | 0.06 | |  |  |  |  |  |
| No, light or moderate | ref | 0.87(0.65,1.16) | | | 0.34 | 0.74(0.55,1.00) | | 0.05 | 0.06 | |  | |  |  |  |  |  |
| Heavy | ref | 1.76(0.74, 4.22) | | | 0.20 | 1.46(0.63, 3.37) | | 0.37 | 0.69 | |  | |  |  |  |  |  |
| ^a^ Trend test was performed using the median value of each diet score tertile as a continuous variable. ^b^ *P* value for interaction was estimated using the likelihood ratio test comparing the model with and without the interaction term of the energy-adjusted dietary garlic consumption and the respective stratification variable. ^c^ Light, moderate, and heavy alcohol consumption are defined as <=6 g/day, > 6–28 g/day for male and > 6–14 g/day for female, and > 28 g/day for male and > 14 g/day for female, respectively. Hazard ratios and 95% confidence intervals of incident colorectal cancer were adjusted for age (<median vs. >=median), sex (male vs. female), race (white, non-Hispanic vs. black, non-Hispanic vs. others), physical activity (none vs. <=1 hour/week vs. >=2 hours/week), diabetes (no vs. yes), cigarette smoking (never vs. current vs. former), BMI (<25 vs. >=25 kg/m2), alcohol consumption (no/light/moderate vs. heavy), and energy from diet (kcal/day), except the stratification factor. | | | | | | | | | | | | |  |  |  |  |  |
|  |  |  |  |  |  |  |  |  |  |  |  |  |  |  |  |  |  |
|  |  |  |  |  |  |  |  |  |  |  |  |  |  |  |  |  |  |
|  |  |  |  |  |  |  |  |  |  |  |  |  |  |  |  |  |  |
|  |  |  |  |  |  |  |  |  |  |  |  |  |  |  |  |  |  |
|  |  |  |  |  |  |  |  |  |  |  |  |  |  |  |  |  |  |
|  |  |  |  |  |  |  |  |  |  |  |  |  |  |  |  |  |  |
|  |  |  |  |  |  |  |  |  |  |  |  |  |  |  |  |  |  |
|  | | | | | | | | | | | | | | | | |  |
|  |  |  |  |  |  |  |  |  |  |  |  |  |  |  |  |  |  |
|  |  |  |  |  |  |  |  |  |  |  |  |  |  |  |  |  |  |
|  |  |  |  |  |  |  |  |  |  |  |  |  |  |  |  |  |  |
| Supplementary Table 5. Sensitivity analyses on the association between quintiles of energy-adjusted garlic consumption (g/day) and overall colorectal cancer incidence. | | | | | | | | | | | | | | | |  |  |
|  | | | Quintiles of energy-adjusted garlic consumption (g/day), HR (95%CI) | | | | | | | | | | | |  |  |  |
| Categories | | | Quintile 1 | Quintile 2 | | | Quintile 3 | | | Quintile 4 | | Quintile 5 | | | *P* trend |  |  |
| Overall CRC | | |  |  | | |  | | |  | |  | | |  |  |  |
| Primary analysis | | | 1 (Ref) | 0.92 (0.73-1.15) | | | 0.70 (0.54-0.91) | | | 0.82 (0.64-1.04) | | 0.85 (0.67-1.06) | | | 0.434 |  |  |
| Excluded participants with extreme energy intake from diet | | | 1 (Ref) | 0.84 (0.67-1.07) | | | 0.67 (0.52-0.87) | | | 0.79 (0.62-1.00) | | 0.82 (0.65-1.03) | | | 0.425 |  |  |
| Excluded participants with history of diabetes | | | 1 (Ref) | 0.92 (0.73-1.17) | | | 0.70 (0.54-0.91) | | | 0.82 (0.64-1.05) | | 0.84 (0.66-1.05) | | | 0.337 |  |  |
| Excluded participants with extreme BMI | | | 1 (Ref) | 0.90 (0.71-1.13) | | | 0.71 (0.54-0.92) | | | 0.81 (0.64-1.04) | | 0.85 (0.68-1.07) | | | 0.48 |  |  |
| Excluded participants within the first 2 years of follow-up | | | 1 (Ref) | 0.92 (0.70-1.21) | | | 0.70 (0.51-0.96) | | | 0.93 (0.70-1.24) | | 0.86 (0.65-1.13) | | | 0.629 |  |  |
| Repeated analysis for participants with unimputed data | | | 1 (Ref) | 0.95 (0.75-1.21) | | | 0.75 (0.57-0.97) | | | 0.84 (0.65-1.07) | | 0.85 (0.67-1.08) | | | 0.333 |  |  |
| Additional adjustment for Healthy Eating Index-2015 | | | 1 (Ref) | 0.92 (0.73-1.16) | | | 0.71 (0.55-0.92) | | | 0.83 (0.65-1.06) | | 0.87 (0.69-1.10) | | | 0.6 |  |  |
|  | | |  |  | | |  | | |  | |  | | |  |  |  |
| Males | | |  |  | | |  | | |  | |  | | |  |  |  |
| Primary analysis | | | 1 (Ref) | 0.94 (0.70-1.25) | | | 0.57 (0.40-0.81) | | | 0.89 (0.65-1.21) | | 0.91 (0.70-1.19) | | | 0.986 |  |  |
| Excluded participants with extreme energy intake from diet | | | 1 (Ref) | 0.87 (0.65-1.17) | | | 0.52 (0.36-0.76) | | | 0.84 (0.61-1.16) | | 0.88 (0.67-1.16) | | | 0.966 |  |  |
| Excluded participants with history of diabetes | | | 1 (Ref) | 0.95 (0.71-1.27) | | | 0.57 (0.40-0.82) | | | 0.88 (0.64-1.20) | | 0.89 (0.68-1.17) | | | 0.84 |  |  |
| Excluded participants with extreme BMI | | | 1 (Ref) | 0.90 (0.68-1.21) | | | 0.56 (0.39-0.80) | | | 0.89 (0.65-1.21) | | 0.91 (0.70-1.19) | | | 0.921 |  |  |
| Excluded participants within the first 2 years of follow-up | | | 1 (Ref) | 0.86 (0.61-1.23) | | | 0.58 (0.37-0.89) | | | 1.01 (0.70-1.45) | | 0.87 (0.63-1.21) | | | 0.929 |  |  |
| Repeated analysis for participants with unimputed data | | | 1 (Ref) | 0.97 (0.73-1.31) | | | 0.61 (0.42-0.88) | | | 0.89 (0.65-1.23) | | 0.91 (0.69-1.19) | | | 0.826 |  |  |
| Additional adjustment for Healthy Eating Index-2015 | | | 1 (Ref) | 0.94 (0.70-1.25) | | | 0.57 (0.40-0.81) | | | 0.89 (0.65-1.21) | | 0.91 (0.70-1.19) | | | 0.986 |  |  |
|  | | |  |  | | |  | | |  | |  | | |  |  |  |
| Females | | |  |  | | |  | | |  | |  | | |  |  |  |
| Primary analysis | | | 1 (Ref) | 0.87 (0.58-1.32) | | | 0.76 (0.49-1.18) | | | 0.73 (0.48-1.11) | | 0.70 (0.45-1.10) | | | 0.16 |  |  |
| Excluded participants with extreme energy intake from diet | | | 1 (Ref) | 0.82 (0.55-1.22) | | | 0.79 (0.51-1.20) | | | 0.73 (0.49-1.10) | | 0.69 (0.45-1.07) | | | 0.15 |  |  |
| Excluded participants with history of diabetes | | | 1 (Ref) | 0.88 (0.58-1.33) | | | 0.76 (0.49-1.19) | | | 0.75 (0.49-1.14) | | 0.70 (0.44-1.10) | | | 0.15 |  |  |
| Excluded participants with extreme BMI | | | 1 (Ref) | 0.88 (0.58-1.33) | | | 0.79 (0.50-1.23) | | | 0.73 (0.48-1.11) | | 0.71 (0.45-1.12) | | | 0.153 |  |  |
| Excluded participants within the first 2 years of follow-up | | | 1 (Ref) | 1.02 (0.63-1.66) | | | 0.79 (0.47-1.34) | | | 0.89 (0.55-1.47) | | 0.82 (0.48-1.40) | | | 0.469 |  |  |
| Repeated analysis for participants with unimputed data | | | 1 (Ref) | 0.92 (0.60-1.40) | | | 0.80 (0.51-1.26) | | | 0.76 (0.50-1.18) | | 0.73 (0.46-1.16) | | | 0.169 |  |  |
| Additional adjustment for Healthy Eating Index-2015 | | | 1 (Ref) | 0.87 (0.58-1.32) | | | 0.76 (0.49-1.18) | | | 0.73 (0.48-1.11) | | 0.70 (0.45-1.10) | | | 0.16 |  |  |
| Extreme values of energy intake are defined as <800 or >4,000 kcal/day for men and <500 or >3,500 kcal/day for women. Extreme values of BMI are defined as the top 99th percentile and the bottom 1st percentile. Hazard ratios and 95% confidence intervals of incident colorectal cancer were adjusted for age (years), sex (male vs. female), race (white, non-Hispanic vs. black, non-Hispanic vs. Hispanic vs. others), physical activity (none vs. <=1 hour/week vs. >=2 hours/week), diabetes (no vs. yes), cigarette smoking (never vs. current vs. former), BMI (kg/m2), alcohol consumption (g/day), and energy from diet (kcal/day). Healthy Eating Index-2015 was treated as the continuous variable in the multivariable Cox regression. | | | | | | | | | | | | | | | |  |  |
|  |  |  |  |  |  |  |  |  |  |  |  |  |  |  |  |  |  |
|  |  |  |  |  |  |  |  |  |  |  |  |  |  |  |  |  |  |
|  |  |  |  |  |  |  |  |  |  |  |  |  |  |  |  |  |  |
|  |  |  |  |  |  |  |  |  |  |  |  |  |  |  |  |  |  |
|  |  |  |  |  |  |  |  |  |  |  |  |  |  |  |  |  |  |
|  |  |  |  |  |  |  |  |  |  |  |  |  |  |  |  |  |  |
